# Automated, open-source, vendor-independent quality assurance protocol based on the Pulseq framework

**DOI:** 10.1007/s10334-025-01247-1

**Published:** 2025-04-24

**Authors:** Qingping Chen, Niklas Wehkamp, Cai Wan, Patrick Hucker, Martin Büchert, Sebastian Littin, Jon-Fredrik Nielsen, Maxim Zaitsev

**Affiliations:** 1 Division of Medical Physics, Department of Radiology, University Medical Center Freiburg, Faculty of Medicine, University of Freiburg, Freiburg, Germany; 2 School of Electrical Engineering, Chongqing University, Chongqing, China; 3 Core Facility MRDAC, Department of Radiology, University Medical Center Freiburg, Faculty of Medicine, University of Freiburg, Freiburg, Germany; 4 Department of Biomedical Engineering, University of Michigan, Ann Arbor, MI USA

**Keywords:** Quality assurance, Open source, Vendor independent, Sequence development, Pulseq

## Abstract

**Objectives:**

Consistent image quality and signal stability are crucial for neuroimaging, particularly fMRI studies that rely on detecting small BOLD signal changes. Regular MR system performance monitoring is essential, especially for longitudinal and multi-site studies. This work aims to establish a robust quality assurance (QA) protocol to enhance data comparability across days, scanner versions, vendors, and sites.

**Materials and methods:**

We implemented an open-source, vendor-independent QA protocol using Pulseq for standardized data acquisition and ISMRMRD/Gadgetron for harmonized image reconstruction, accompanied by an automated post-processing pipeline to evaluate structural and temporal image quality. The protocol was thoroughly tested on three Siemens 3T scanners with different software versions at one site, and one GE 3T scanner at another site. The test was repeated on an fBIRN phantom for at least 4 days.

**Results:**

The vendor-independent protocol produced image quality comparable to a closely matched vendor-based protocol. It showed similar day-to-day repeatability to the vendor-based protocol across the Siemens scanners and high inter-day repeatability on the GE scanner.

**Conclusion:**

We successfully developed and implemented an open-source, vendor-independent QA protocol, accompanied by an automated post-processing pipeline. The results demonstrate the feasibility and repeatability of the protocol across different days, system versions, vendors, and sites.

## Introduction

Neuroimaging research relies heavily on consistent image quality and magnetic resonance (MR) signal stability over extended periods of time. This applies to both anatomical imaging and functional magnetic resonance imaging (fMRI). In particular, most fMRI studies are based on blood-oxygen-level-dependent signal changes caused by neuronal activity, which are only a small fraction of the original signal intensity [1]. To accurately measure such subtle signal changes, an MR system must have an intrinsic temporal signal fluctuation level that is much lower than the expected signal changes. Therefore, regular monitoring of MR system performance is crucial to ensure that the instrument maintains an acceptable condition, thereby enhancing the success rate of neuroimaging studies.

Quality assurance (QA) is particularly important in longitudinal and/or multi-site studies, where data are collected from a group of subjects over time and/or across different sites. Regular monitoring of scanner performance over time and/or at different sites is essential to identify and, if possible, control for intrinsic differences (e.g., due to model-specific hardware and certain software variations) and changes in the scanner performance (e.g., due to software and/or hardware upgrades and gradual component aging). Failure to account for these factors may result in unexplained discrepancies in the data [2]. Therefore, robust QA protocols are crucial for establishing consistent data standards and ensuring data comparability over time and across scanners, which is critical for data pooling to enhance statistical power. Many QA protocols have been proposed to monitor the performance of MR scanners [2–8], and most of them tried to harmonize measurement protocols as much as possible using vendor-provided pulse sequences and image reconstruction tools.

However, the intricate details of data acquisition (e.g., details of the fat suppression module, actual gradient strength and slew rate utilization, spoiler amplitude and placement, and phase cycling) and image reconstruction (e.g., image smoothing filter, regridding, and denoising) are difficult to harmonize due to the heterogeneity of imaging hardware and reconstruction software. This variability poses a substantial challenge for QA comparisons across vendors and sites. For example, fat suppression techniques vary across vendors, with GE defaulting to a spectral-spatial pulse and Siemens utilizing a fat-saturation pulse. In addition, image smoothing defaults differ: Siemens scanners typically do not apply smoothing, whereas GE employs a Fermi k-space filter. These variations may notably impact the absolute values of QA metrics, as detailed by Glover et al. in their recommendations for multi-center fMRI studies [4]. We hypothesize that full access to the data acquisition protocol, image reconstruction algorithms, and post-processing pipeline within a QA framework would enable the MR community to assess scanner performance objectively and trace observed differences to their underlying causes. This underscores the need for standardized QA protocols covering data acquisition, image reconstruction, and post-processing across diverse scanner-specific environments.

Given the above, in this work, we implemented an open-source, vendor-independent QA protocol for data acquisition and image reconstruction, accompanied by an automated post-processing pipeline. The protocol is based on our previously proposed Pulseq framework [9, 10], which we extended and integrated with Siemens’ “Image Calculation Environment” (ICE) platform and Gadgetron [11] using the vendor-independent ISMRMRD [12] data format. This QA protocol enables the assessment of structural quality (e.g., image uniformity) and temporal quality (e.g., temporal stability) of MR systems based on the recommendations of the American College of Radiology (ACR) as well as the American Association of Physicists in Medicine (AAPM) [13, 14] and the Function Biomedical Informatics Research Network (fBIRN) [4], respectively. It has been tested on three Siemens 3T scanners with substantially different hardware configurations and software versions at one site and one GE 3T scanner at another site. The tests were performed repeatedly on an fBIRN gel-filled phantom over at least 4 different days.

## Materials and methods

### Data acquisition

Based on the Pulseq framework, a spin-echo (SE) sequence was implemented to measure structural quality metrics [13]; and an echo-planar imaging (EPI) sequence was implemented to assess temporal quality metrics, in accordance with a previously published protocol [2]. Siemens product-based SE and EPI protocols were configured to closely match the corresponding Pulseq-based protocols. The sequence parameters are listed in Table 1.

**Table 1:** Parameters of Siemens product-based and Pulseq-based SE and EPI sequences

	FOV (mm^3^)	Slice thick./gap (mm/mm)	Matrix size	TR/TE/TA (ms/ms/min:s)	Readout BW (Hz/Px)	Echo spacing (ms)	Exc./ref. FA (°/°)	#rep
SE								
Product	250*250*105	5/5	256*256*11	500/20/4:20	195	-	90/180	2
Pulseq	250*250*105	5/5	256*256*11	500/20/**4:16**	195	-	90/180	2
EPI								
Product	220*220*134	4/1	64*64*27	2000/22/6:44	1954	0.58	90/–	200
Pulseq	220*220*134	4/1	64*64*27	2000/**24**/**6:40**	**1923**	0.58	90/–	200

For all sequences, the slice orientation is “Transversal,” and the phase-encoding direction is from anterior to posterior (“A >> P” on Siemens scanners). Differences between the product-based and Pulseq-based sequences are highlighted in bold

SE = spin-echo; EPI = echo-planar imaging; FOV = field of view; slice thick./gap = slice thickness/gap; TR/TE/TA = repetition time/echo time/total acquisition time; readout BW = readout bandwidth; exc./ref. FA = excitation/refocusing flip angle; #rep = number of repetitions

The QA protocol consists of one SE acquisition followed by three EPI scans. The first two EPI scans let the gradient coils reach a temperature equilibrium to reduce magnetic field drifts in the subsequent runs. The third identical EPI scan and the SE acquisition generate data used to calculate the QA metrics. A homogeneous, gel-filled fBIRN phantom (EZfMRI, Chicago, IL, USA) with *T*1/*T*2 values of 806.9 ± 2.4 ms/81.7 ± 0.5 ms was scanned using both vendor-based and Pulseq-based QA protocols on three Siemens 3T scanners: Trio TIM, Prisma.Fit, and Cima.X (SIEMENS Healthineers AG, Forchheim, Germany) in Freiburg, Germany, which were manufactured over a span of 17 years. Note that the fat-saturation module in the EPI sequence for Trio was modified due to hardware limitations. All measurements at Freiburg were performed on the same fBIRN phantom. Images for *T*1 fitting were acquired using a product turbo spin-echo sequence with an inversion recovery pulse and the following parameters: repetition time = 4000 ms, echo train length = 4, and inversion recovery times = {50, 150, 300, 450, 600, 750, 900, 1050, 1200, 1350, 1500, 2200, 3000} ms. Images for *T*2 fitting were obtained using a product SE sequence with the following parameters: repetition time = 3500 ms and echo times = {7.5, 15, 30, 45, 60, 75, 90, 130, 200, 250} ms. Both measurements were conducted on the Siemens Prisma.Fit 3T scanner, and the acquired DICOM images, along with post-processing scripts, are available online [15]. The Pulseq-based QA protocol was performed on another copy of the fBIRN phantom from the same manufacturer (EZfMRI, Chicago, IL, USA) on a GE 3T scanner: UHP (General Electric Healthcare, Waukesha, WI, USA) at the University of Michigan, USA [16]. The technical parameters of the four scanners and the used coils are detailed in Table 2. The QA measurements were repeated over a period of 15 days: for ten repetitions on Trio, five repetitions on Prisma.Fit, five repetitions on Cima.X, and four repetitions on UHP.

**Table 2:** Technical parameters of the four scanners and the used coils

Site	Vendor	Hardware model	Software version	Coil
Freiburg	Siemens	Trio TIM	VB19A	12-channel head coil (with the 4-channel “CP mode”)
		Prisma.Fit	XA60A	20-channel head coil
		Cima.X	XA61A	20-channel bioMatrix head coil
Michigan	GE	UHP	UHP	32-channel head coil

The fBIRN phantom was placed in the scanner room for at least several hours before the measurements to allow it to reach room temperature. All lights in the room (including ceiling lights and scanner bore lighting) were turned off, and the bore ventilation was deactivated prior to the measurements. The fBIRN phantom was securely positioned in the head coil using foam padding, maintaining a consistent distance from the patient table across different days. The field of view was carefully adjusted to ensure the phantom was centrally aligned. Automatic standard shim was performed before acquiring the QA data, using the “Standard” shim mode on the Siemens scanners and the “GE Linear Autoshim” on the GE scanner.

### Image reconstruction

**Fig. 1:**
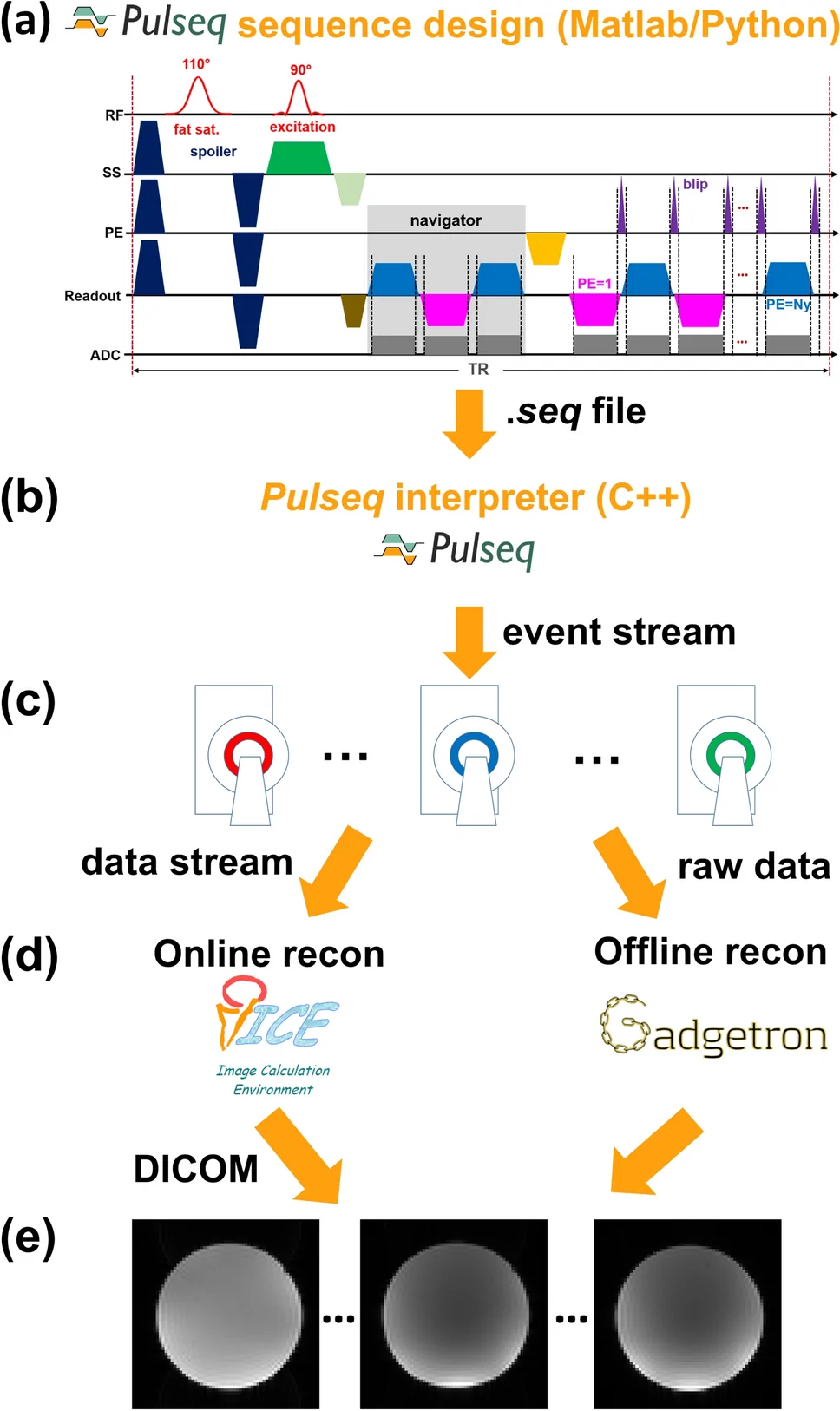
Schematic representation of the data acquisition and image reconstruction workflow. **a** Diagram of the EPI sequence designed using Pulseq. **b** The Pulseq interpreter loads the*.seq* file and streams events to scanners. **c** Data acquisition is conducted on various MR scanners. **d** Raw data are exported for offline reconstruction using Gadgetron. In addition, Siemens data are streamed to the ICE pipeline for online reconstruction. **e** ICE transmits DICOM images directly to the MR host computer immediately after measurements. Gadgetron facilitates cross-model and cross-vendor image reconstruction by utilizing the ISMRMRD data format for seamless compatibility

Image reconstruction was conducted using our proposed data acquisition and image reconstruction workflow [9, 10], as illustrated in Fig. 1. This workflow integrates Pulseq with the Siemens ICE platform and open-source, vendor-independent Gadgetron software [11]. All data were converted into the vendor-independent ISMRMRD [12] data format and reconstructed offline using Gadgetron. In addition, Siemens data were reconstructed online within the Siemens-integrated ICE environment. No scanner-integrated image reconstruction was implemented for the Pulseq-based data on the GE platform. Detailed instructions on image reconstruction are available online [15, 17].

### Post-processing

An automated post-processing pipeline was developed to mask the SE and echo-planar images and perform structural and temporal quality analysis. All related source code is readily available online [15, 17].

#### Automated masking

**Fig. 2:**
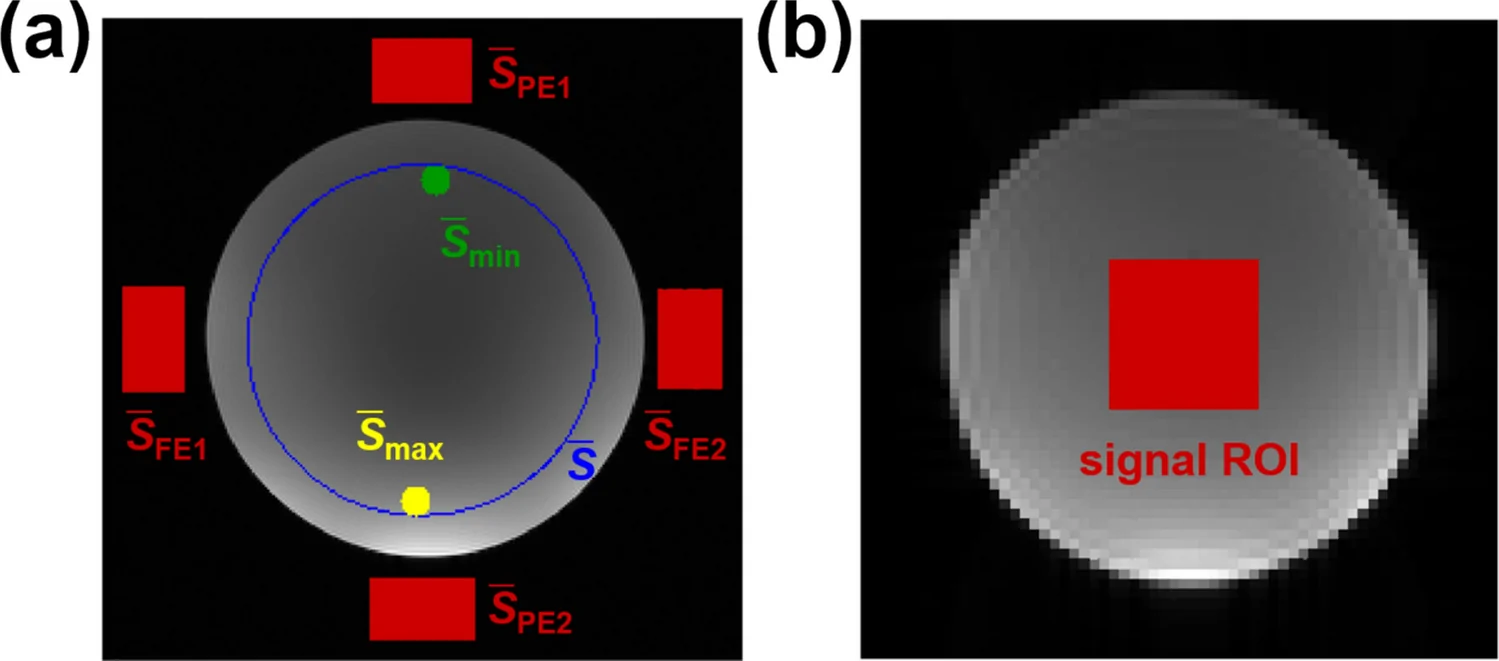
Automated masking. **a** Two small circular ROIs (~ 1 cm^2^ each), representing regions of minimal (green) and maximal (yellow) mean signal intensities, are placed within a large circular signal ROI (blue outline). The large ROI is centrally aligned with the phantom and has a diameter equal to 80% of the phantom’s diameter. In addition, four background rectangular ROIs in red (~ 10 cm^2^ each) are positioned in the SE image. **b** A square ROI containing 15 × 15 pixels (red) is centrally positioned on the phantom in the echo-planar image

The circular edge of the phantom in the SE and echo-planar images is automatically detected, and this information is used to determine the center position of the phantom. The regions of interest (ROIs) used for calculating QA metrics are automatically masked in the SE and echo-planar images based on the fitted center position. As illustrated in Fig. 2a, a large circular ROI, with a diameter corresponding to 80% of the phantom’s diameter, is centered on the phantom in the SE image to determine the mean signal intensity ($\overline{S}$). Two smaller circular ROIs (~ 1 cm^2^ each) within the large ROI are used to measure the minimal ($\overline{S}_{\min }$) and maximal ($\overline{S}_{\max }$) mean signal intensities. Four rectangular ROIs (~ 10 cm^2^ each) outside the phantom are positioned to measure background mean signal intensities along the frequency-encoding ($\overline{S}_{{{\mathrm{FE1}}}}$, $\overline{S}_{{{\mathrm{FE2}}}}$) and phase-encoding ($\overline{S}_{{{\mathrm{PE1}}}}$, $\overline{S}_{{{\mathrm{PE2}}}}$) directions. As depicted in Fig. 2b, a 15 × 15 pixel ROI is centered on the phantom in the echo-planar image for temporal quality analysis.

#### Structural quality analysis

The structural quality analysis assesses the quality of structural or anatomical images produced by an MR system. The evaluation is conducted on the sixth slice of the SE images, using data from either the first repetition or two repetitions, as recommended by the ACR and AAPM guidelines [13, 14]. The relevant metrics are outlined below.

##### Percent signal ghosting (PSG)

Ghosting in structural imaging typically results from signal instabilities across multiple repetition time (TR) periods during a scan. This artifact, commonly observed in the PE direction, can obscure image details and is particularly noticeable in low-signal regions. To quantify ghosting, the PSG is calculated as the difference in signal intensity between the background ROIs in the FE direction ($\overline{S}_{{{\mathrm{FE1}}}}$ and $\overline{S}_{{{\mathrm{FE2}}}}$) and those in the PE direction ($\overline{S}_{{{\mathrm{PE1}}}}$ and $\overline{S}_{{{\mathrm{PE2}}}}$), relative to the mean signal intensity of the large circular signal ROI ($\overline{S}$):

1 $$\begin{array}{*{20}c} {{\mathrm{PSG}} = 100 \cdot \left| { \frac{{\left( {\overline{S}_{{{\mathrm{FE1}}}} + \overline{S}_{{{\mathrm{FE2}}}} } \right) {-} \left( {\overline{S}_{{{\mathrm{PE1}}}} + \overline{S}_{{{\mathrm{PE2}}}} } \right)}}{{2\overline{S}}}} \right|.} \\ \end{array}$$

##### Percent image uniformity (PIU)

Image uniformity reflects the ability of an MR system to represent uniform areas of a phantom with a consistent intensity. Deviations from uniformity often arise from radio-frequency or static magnetic field inhomogeneities, or insufficient eddy current compensation. PIU serves as a quantitative measure of image uniformity and is calculated as

2 $$\begin{array}{*{20}c} {{\mathrm{PIU}} = 100 \cdot \left[ {1 {-} \frac{{\left( {\overline{S}_{\max } {-} \overline{S}_{\min } } \right)}}{{\left( {\overline{S}_{\max } + \overline{S}_{\min } } \right)}}} \right].} \\ \end{array}$$

##### Signal-to-noise ratio (SNR)

SNR is a key parameter that reflects the clinical utility of an MR image and serves as a sensitive indicator of both hardware performance and image reconstruction efficiency. In accordance with the NEMA standards [18], two methods are used in this work to calculate SNR. The first method calculates SNR from a single magnitude-reconstructed image obtained from the first repetition, expressed as

3 $$\begin{array}{*{20}c} {{\mathrm{SNR}}_{1} = \sqrt {\left( {4 {-} \pi } \right)/2} \cdot \frac{{\overline{S}}}{{{\mathrm{SD}}_{1} }},} \\ \end{array}$$

where $\sqrt {\left( {4 {-} \pi } \right)/2}$ is the correction factor [19]; $\overline{S}$ is the mean signal intensity from the large circular ROI; and ${\mathrm{SD}}_{{1}}$ is the standard deviation of the pixel values from the two background ROIs along the FE direction.

The second method involves the subtraction ($I_{{{\mathrm{diff}}}} = I_{1} {-} I_{2}$) of two magnitude images obtained from the first ($I_{1}$) and the second ($I_{2}$) SE repetitions. This SNR is then determined by

4 $$\begin{array}{*{20}c} {{\mathrm{SNR}}_{2} = \sqrt 2 \cdot \frac{{\overline{S}}}{{{\mathrm{SD}}_{2} }},} \\ \end{array}$$

where $\sqrt {2}$ is the correction factor; $\overline{S}$ is the mean ROI signal intensity from the first repetition; and ${\mathrm{SD}}_{{2}}$ is the standard deviation of the pixel values within the large circular ROI on $I_{{{\mathrm{diff}}}}$.

Both methods carry inherent risks. In the first method, subtle, invisible artifacts can distort the ${\mathrm{SD}}_{{1}}$ measurement, and image post-processing may further alter ${\mathrm{SD}}_{{1}}$. In addition, when acquiring images at high bandwidths, non-uniformities in the receiver subsystem’s frequency response can lead to differing noise statistics across the image [20]. In the second method, system drift can affect the determination of image noise statistics when subtracting two sequentially acquired images. Temporal instability in the MR system may introduce artifacts into the subtracted image, thereby increasing ${\mathrm{SD}}_{{2}}$. The ratio between the two SNR values is calculated as ${\mathrm{SNR}}_{{{\mathrm{ratio}}}} {\text{ = SNR}}_{{1}} /{\mathrm{SNR}}_{{2}}$. Ideally, ${\mathrm{SNR}}_{{{\mathrm{ratio}}}}$ should equal 1, and in this work, a range of [0.9, 1.1] was considered acceptable. A significant deviation from 1 in ${\mathrm{SNR}}_{{{\mathrm{ratio}}}}$ suggests that the noise statistics may be compromised in one or both methods.

#### Temporal quality analysis

Signal stability over time is essential for prolonged EPI scans. In this study, temporal quality analysis assesses this aspect of MR system performance. The analysis is conducted on the central slice of the third EPI scan across 198 volumes, with the first two volumes discarded, in accordance with the fBIRN guidelines [4]. The associated analyses and metrics are detailed below.

##### Signal image

The signal image is derived by calculating the pixel-by-pixel average across the 198 echo-planar images.

##### Temporal fluctuation noise image

A second-order polynomial detrending is applied to the time series of each pixel across the 198 volumes. The temporal fluctuation noise image reflects the pixel-by-pixel standard deviation of the detrended time series.

##### Signal-to-fluctuation-noise ratio (SFNR)

The SFNR is one of the critical parameters to estimate the signal stability over time. The signal image and the temporal fluctuation noise image are divided pixel by pixel to create the SFNR image. The summary SFNR value is the average SFNR across the pixels within the signal ROI (Fig. 2b).

##### Percent fluctuation

Percent fluctuation is another parameter used to assess temporal stability. First, the average signal intensity within the signal ROI is calculated for each volume, resulting in a time series of 198 values. This time series is then detrended using a second-order polynomial fitting algorithm. The percent fluctuation is calculated as $100 \times {\mathrm{SD}}/M$, where ${\mathrm{SD}}$ represents the standard deviation of the detrended time series, and M is the mean of the spatially averaged time series.

##### Drift

Temperature fluctuations during the EPI scans can cause variations in magnetic field strength, leading to signal drift over time. This drift is calculated as $100 \times { }\left( {{\mathrm{trend}}_{{{\mathrm{max}}}} - {\mathrm{trend}}_{{{\mathrm{min}}}} } \right)/M$, where ${\mathrm{trend}}_{{{\mathrm{max}}}}$ and ${\mathrm{trend}}_{{{\mathrm{min}}}}$ represent the maximum and minimum values of the second-order polynomial trend, respectively; and M is the mean of the spatially averaged time series.

##### Temporal-spatial signal correlation analysis

It is also known as Weisskoff analysis [21], provides an additional method for assessing scanner stability. This approach is based on the assumption that scanner instabilities increase inter-pixel correlation, likely due to the low-spatial-frequency nature of these instabilities. In the absence of scanner instability, temporal fluctuation is expected to arise solely from the thermal noise of the scanner and the phantom, ignoring minimal correlation resulting from imperfections in the point spread function. Consequently, under the assumption that the temporal variance of individual pixels is uncorrelated, the temporal fluctuation of an averaged signal from an ROI is expected to decrease as the number of pixels within the ROI increases. This relationship can be formulated as

5 $$\begin{array}{*{20}c} {{\mathrm{SD}}\left( N \right) = \sqrt {\frac{{\mathop \sum \nolimits_{i}^{V} \left[ {\frac{{\mathop \sum \nolimits_{j}^{{N^{2} }} R_{i,j} }}{{N^{2} }} - \overline{R}} \right]^{2} }}{V - 1}} = \frac{{{\mathrm{SD}}\left( 1 \right)}}{N},} \\ \end{array}$$

where V = 198 is the number of volumes; *N* is the side length of the square ROI; and $R_{i,j}$ represents the value (the *i*th volume and the *j*th pixel) of the residual, which is the difference between the spatially averaged time series and its second-order polynomial trend; $\overline{R} = \frac{{\mathop \sum \nolimits_{i}^{V} \left( {\mathop \sum \nolimits_{j}^{{N^{2} }} R_{i,j} } \right)}}{{V \cdot N^{2} }}$ is the average value of the residual. SD(*N*) and SD(1) refer to the temporal fluctuations of an ROI with side lengths of *N* and 1, respectively. Thus, for a square *N* × *N* pixel ROI, a plot of log(SD(*N*)) versus log(*N*) should follow a declining straight line. In practice, as *N* increases, the reduction in SD(*N*) flattens out towards a plateau and becomes independent of *N*. This occurs because system instabilities introduce low-spatial-frequency correlations in the image, causing the pixels to lose their statistical independence. By comparing the actual temporal fluctuation, SD_actual_(*N*), with the theoretically approachable value, $\frac{{{\mathrm{SD}}_{{{\mathrm{actual}}}} \left( {1} \right)}}{N}$, the impact of these fluctuations can be assessed. Example Weisskoff plots are presented in “Results”.

##### Radius of decorrelation (RDC)

The RDC measures the ROI side length at which statistical independence of the pixels is lost. A higher RDC indicates better scanner stability. It is calculated as $\frac{{{\mathrm{SDactual(1)}}/M_{{1}} }}{{{\mathrm{SDactual(}}N{)}/M_{N} }}$, where ${\mathrm{SDactual(1)}}$ and ${\mathrm{SDactual(N)}}$ are the actual temporal fluctuations of the ROIs with side lengths of 1 and *N*, respectively (Eq. 5); $M_{1}$ and $M_{N}$ are the mean values of the spatially averaged time series for the ROIs with side lengths of 1 and *N*, respectively.

The complete summary of all QA metrics can be found in Table 4 in the Appendix.

### Statistical analysis

The means and standard deviations of each scanner’s structural and temporal metrics over different days were calculated. The ratios of standard deviations to means were then used to estimate the repeatability of the QA protocol.

## Results

**Fig. 3:**
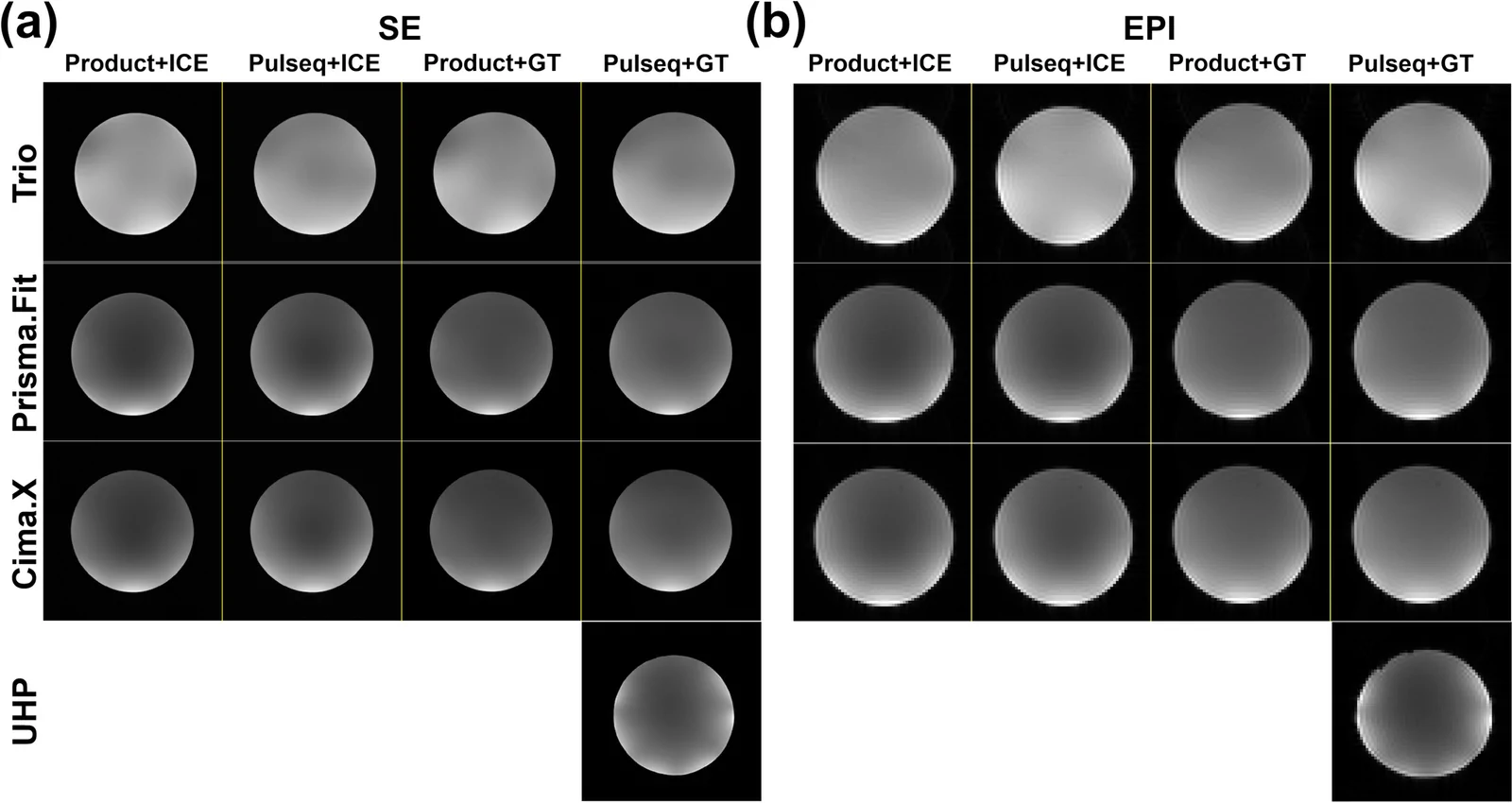
Representative images for QA analysis. **a** SE images from the first repetition, used for structural quality analysis. **b** Signal images from the third EPI scan, used for temporal quality analysis. Product = vendor-provided sequence; ICE = Siemens’ integrated reconstruction platform; GT = Gadgetron; SE = spin-echo; EPI = echo-planar imaging

Figure 3 presents the normalized vendor-based and Pulseq-based SE and echo-planar images reconstructed using ICE and Gadgetron from three Siemens scanners and one GE scanner. The Pulseq-generated images are largely consistent with the corresponding product-based images across all three Siemens scanners. Gadgetron demonstrates reconstruction performance comparable to Siemens’ integrated ICE software. Visual inspection suggests that the quality of the Pulseq-based images from the GE platform is similar to that of the corresponding Siemens images.

**Fig. 4:**
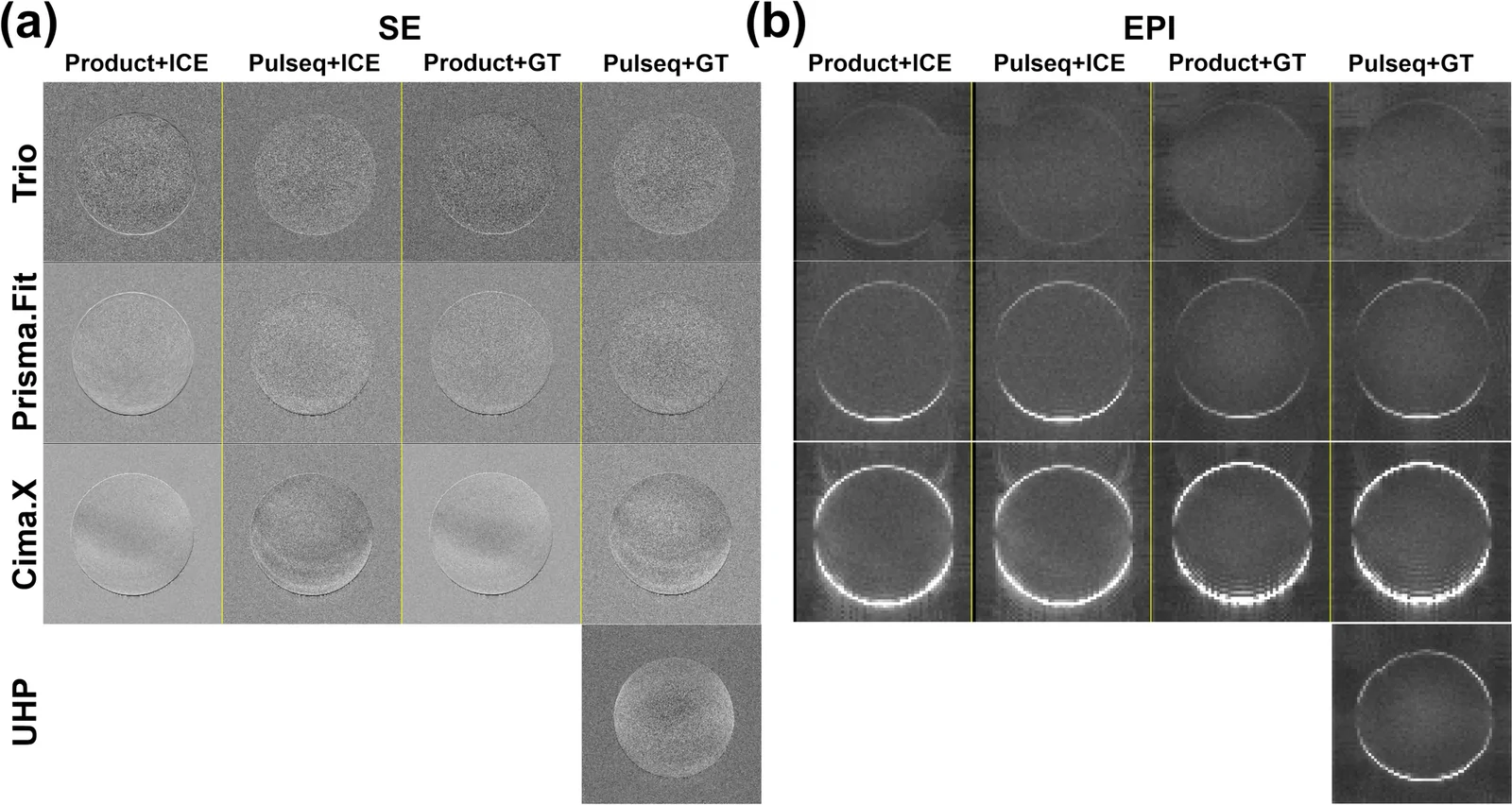
Representative noise images for QA analysis. **a** Image differences between the first and second repetitions of the SE acquisition. **b** Temporal fluctuation noise images from the third EPI scan. Each image is thresholded at 3*$\overline{{{\mathrm{STD}}}}_{{{\mathrm{ROI}}}}$, where $\overline{{{\mathrm{STD}}}}_{{{\mathrm{ROI}}}}$ is the average pixel value within the square signal ROI of the image. Product = vendor-provided sequence; ICE = Siemens’ integrated reconstruction platform; GT = Gadgetron; SE = spin-echo; EPI = echo-planar imaging

Figure 4a illustrates the image difference between the first and second SE repetitions ($I_{{{\mathrm{diff}}}}$), which is used to calculate ${\mathrm{SNR}}_{{2}}$. The image differences from Cima.X exhibit prominent ghosting artifacts along the PE direction, likely due to inconsistencies in PE steps between different TRs. Figure 4b displays the temporal fluctuation noise images obtained from the third EPI scan. Cima.X shows a higher level of ghosting in the PE direction compared to the other three scanners, likely resulting from scanner instability during the EPI acquisition. In both Fig. 4a, b, the Pulseq-based (Gadgetron-based) protocol exhibits noise and artifact behaviors similar to those of the product-based (ICE-based) protocol, indicating the reliability of the proposed vendor-independent QA protocol.

Table 3 presents the summary statistics of QA metrics derived from both product-based and Pulseq-based acquisitions, with reconstructions performed using ICE and Gadgetron, collected over multiple days across four scanners. For most QA metrics, the ratios of standard deviations to means are below 10%, except for PSG and Drift. PSG, which is sensitive to intra-scan signal instabilities, exhibits large inter-day variability across all scanners; however, it consistently maintains a relatively low mean value of less than 2.4 ‰. In addition, some temporal signal drifts, as well as the product-based, ICE-based RDCs and percent fluctuations from Prisma.Fit, exhibit slightly higher inter-day variability. This variability may be attributed to fluctuating temperature levels on different days, including changes in the room temperature and gradient coil temperature, which can be influenced by preceding measurement slots in the daily scanner schedule. Furthermore, all SNR ratios fall within the acceptable range of [0.9, 1.1], except for the product-based, ICE-based SNR ratio on Prisma.Fit (0.85 ± 0.02), which is slightly below the acceptable range. This implies that the SNR calculations in the post-processing pipeline are generally reliable. Overall, these observations indicate that the proposed QA protocol demonstrates good repeatability across different days.

**Table 3:** Summary statistics of QA metrics

Scanner	Recon	Seq	PSG (‰)	PIU (%)	SNR1/SNR2/Ratio	SFNR	RDC	Fluc. (‰)	Drift (%)
Trio (Siemens)	ICE	Product	0.29 ± 0.13 ***(46%)***	85.5 ± 0.8 *(1%)*	144 ± 8/153 ± 12/0.94 ± 0.05 *(6%/8%/5%)*	409 ± 12 *(3%)*	4.10 ± 0.19 *(5%)*	0.61 ± 0.02 *(3%)*	0.41 ± 0.05 ***(13%)***
		Pulseq	0.45 ± 0.20 ***(45%)***	84.8 ± 1.0 *(1%)*	125 ± 8/124 ± 13/1.02 ± 0.04 *(7%/****11%****/4%)*	419 ± 12 *(3%)*	3.95 ± 0.23 *(6%)*	0.62 ± 0.03 *(5%)*	0.79 ± 0.04 *(5%)*
	GT	Product	0.23 ± 0.12 ***(50%)***	82.7 ± 0.7 *(1%)*	123 ± 4/133 ± 4/0.92 ± 0.02 *(4%/3%/2%)*	399 ± 12 *(3%)*	4.24 ± 0.28 *(6%)*	0.62 ± 0.02 *(3%)*	0.41 ± 0.05 ***(13%)***
		Pulseq	0.48 ± 0.20 ***(41%)***	81.0 ± 0.8 *(1%)*	123 ± 4/120 ± 4/1.02 ± 0.03 *(3%/3%/3%)*	410 ± 12 *(3%)*	3.97 ± 0.31 *(8%)*	0.62 ± 0.03 *(5%)*	0.86 ± 0.05 *(6%)*
Prisma.Fit (Siemens)	ICE	Product	1.21 ± 0.40 ***(33%)***	71.0 ± 0.1 *(0%)*	208 ± 4/244 ± 3/0.85 ± 0.02 *(2%/1%/2%)*	481 ± 5 *(1%)*	7.28 ± 0.97 ***(13%)***	0.31 ± 0.04 ***(12%)***	0.21 ± 0.03 ***(12%)***
		Pulseq	1.35 ± 0.25 ***(18%)***	71.2 ± 0.1 *(0%)*	151 ± 3/157 ± 2/0.96 ± 0.01 (2%/1%/1%)	500 ± 4 *(1%)*	6.58 ± 0.27 *(4%)*	0.33 ± 0.02 *(5%)*	0.23 ± 0.04 ***(19%)***
	GT	Product	1.27 ± 0.29 ***(23%)***	77.5 ± 0.2 *(0%)*	154 ± 3/168 ± 1/0.92 ± 0.02 *(2%/0%/2%)*	458 ± 4 *(1%)*	7.31 ± 0.92 ***(13%)***	0.32 ± 0.04 ***(11%)***	0.22 ± 0.03 ***(14%)***
		Pulseq	1.19 ± 0.20 ***(17%)***	77.9 ± 0.2 *(0%)*	137 ± 3/144 ± 1/0.95 ± 0.02 *(2%/1%/2%)*	484 ± 6 *(1%)*	7.28 ± 0.29 *(4%)*	0.33 ± 0.02 *(4%)*	0.31 ± 0.02 *(7%)*
Cima.X (Siemens)	ICE	Product	1.43 ± 0.47 ***(33%)***	70.4 ± 0.3 *(0%)*	167 ± 2/168 ± 10/1.00 ± 0.08 *(1%/6%/8%)*	431 ± 4 *(1%)*	3.19 ± 0.11 *(4%)*	0.77 ± 0.03 *(4%)*	0.41 ± 0.03 *(8%)*
		Pulseq	2.23 ± 0.82 ***(37%)***	70.6 ± 0.2 *(0%)*	148 ± 3/146 ± 2/1.01 ± 0.03 *(2%/1%/3%)*	449 ± 1 *(0%)*	2.95 ± 0.29 *(10%)*	0.79 ± 0.05 *(7%)*	0.44 ± 0.08 ***(18%)***
	GT	Product	1.43 ± 0.48 ***(34%)***	72.0 ± 0.7 *(1%)*	158 ± 3/155 ± 9/1.02 ± 0.08 *(2%/6%/8%)*	410 ± 4 *(1%)*	3.25 ± 0.16 *(5%)*	0.79 ± 0.02 *(2%)*	0.44 ± 0.03 *(8%)*
		Pulseq	2.36 ± 0.93 ***(40%)***	71.7 ± 0.7 *(1%)*	140 ± 3/136 ± 4/1.03 ± 0.05 *(2%/3%/5%)*	426 ± 3 *(1%)*	2.93 ± 0.19 *(7%)*	0.86 ± 0.05 *(6%)*	0.46 ± 0.07 ***(16%)***
UHP (GE)	GT	Pulseq	0.59 ± 0.46 **(78%)**	75.4 ± 0.5 (1%)	154 ± 4/151 ± 2/1.02 ± 0.03 (3%/2%/3%)	408 ± 1 (0%)	5.58 ± 0.44 (8%)	0.51 ± 0.03 (5%)	0.38 ± 0.03 (9%)

The means and standard deviations (SDs) across different days are listed as mean ± SD. The ratios of standard deviations to means are given in the brackets in italics. The ratios larger than 10% are highlighted in bold italics

Recon. = reconstruction; Seq. = sequence; PSG = percent signal ghosting; PIU = percent image uniformity; SNR1 = SNR based on the first repetition; SNR2 = SNR based on two repetitions; Ratio = the ratio between SNR1 and SNR2; SFNR = signal to fluctuation noise ratio; RDC = radius of decorrelation; Fluc. = percent fluctuation; ICE = Siemens’ “image calculation environment”; GT = Gadgetron

**Fig. 5:**
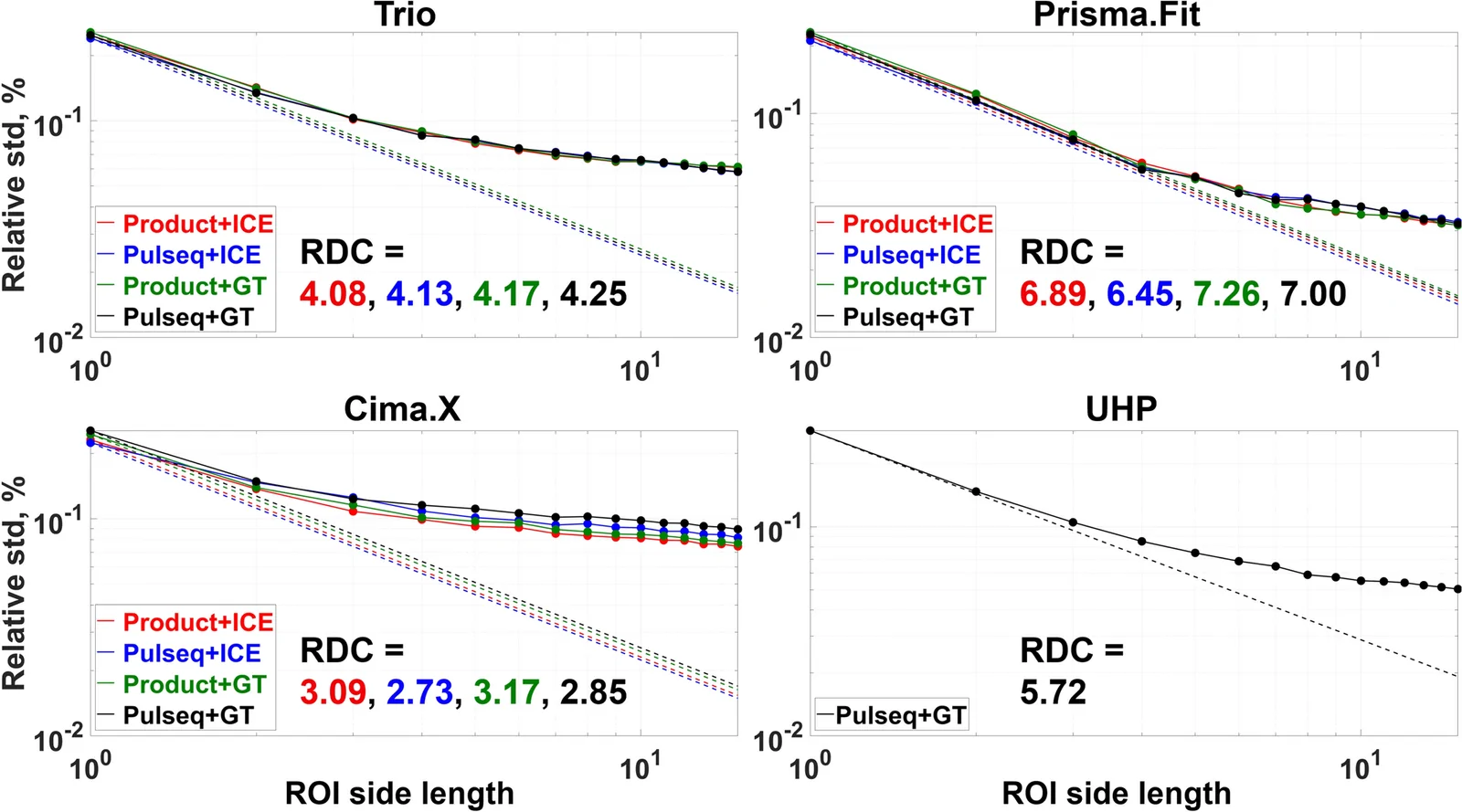
The Weisskoff analysis of representative EPI data acquired from four scanners on a single day. The horizontal axis represents the ROI side length (ranging from 1 to 15, with ROI dimensions from 1 × 1 to 15 × 15). The vertical axis shows the relative standard deviations ($100 \times {\mathrm{SDactual(}}N{)}/M_{N}$) corresponding to the incrementally increasing ROI side length, N. Both axes are presented on a logarithmic scale. The dashed line represents the theoretical expectation, where the relative standard deviation is predicted to decrease linearly with the increasing ROI side length. The solid curve depicts the actual decline, with dots representing the acquired data points. GT = Gadgetron

Figure 5 presents the Weisskoff analysis of representative EPI data acquired from four scanners on a single day. The Weisskoff curves and resulting RDC values generated by the Pulseq-based (Gadgetron-based) protocol align well with those obtained using the corresponding product-based (ICE-based) protocol on the three Siemens scanners. This indicates that the open-source, vendor-independent QA protocol performs comparably to the closely matched scanner-integrated protocol in assessing scanner stability through the Weisskoff plots. Among the four scanners, Prisma.Fit yields the best performance based on these observations, followed by UHP.

**Fig. 6:**
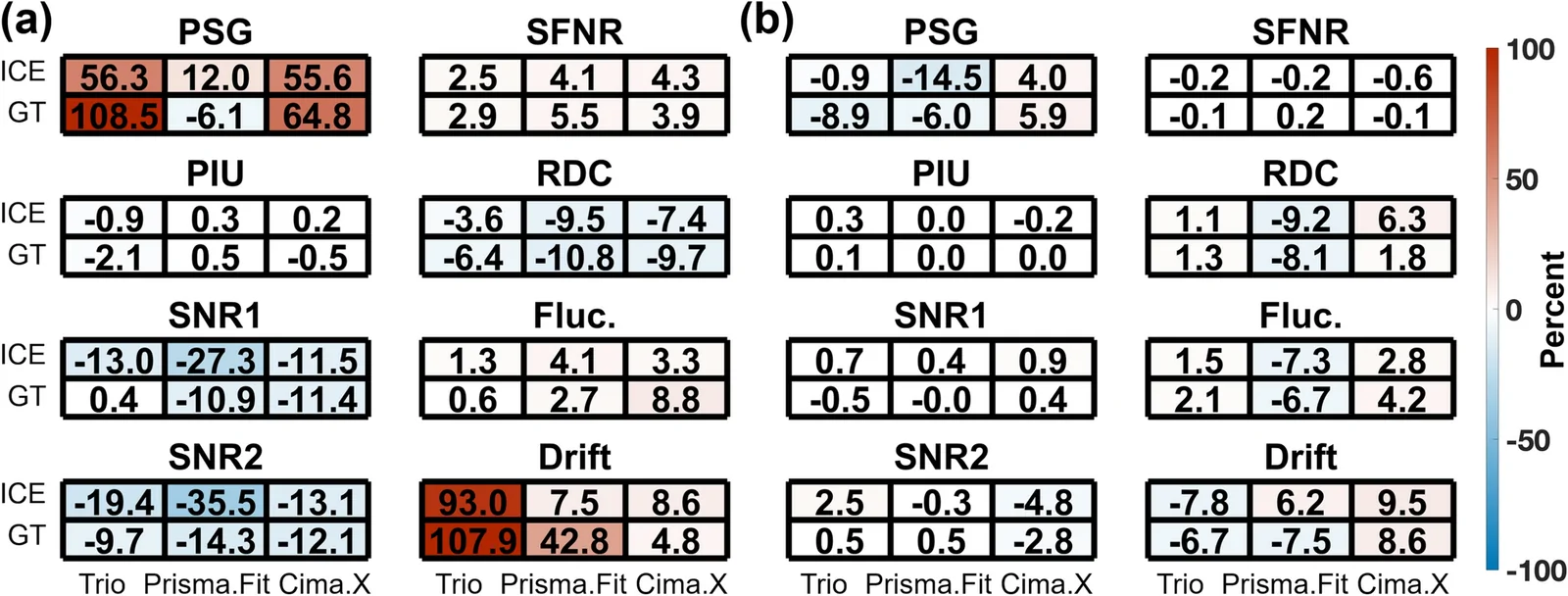
Comparison of QA performance between product-based and Pulseq-based protocols. **a** The difference in mean QA metrics between the two protocols, calculated as $100 \times { }\frac{{{\mathrm{mean}}_{{{\mathrm{Pulseq}}}} { } - {\text{ mean}}_{{{\mathrm{Product}}}} }}{{{\text{ mean}}_{{{\mathrm{Product}}}} }}$, for both ICE and Gadgetron reconstructions on the three Siemens scanners. **b** The difference in relative standard deviation, calculated as $100 \times { }\left( {\frac{{{\mathrm{STD}}_{{{\mathrm{Pulseq}}}} }}{{{\mathrm{mean}}_{{{\mathrm{Pulseq}}}} }} - { }\frac{{{\mathrm{STD}}_{{{\mathrm{Product}}}} }}{{{\mathrm{mean}}_{{{\mathrm{Product}}}} }}} \right)$, for both ICE and Gadgetron reconstructions on the Siemens scanners. PSG = percent signal ghosting; PIU = percent image uniformity; SNR1 = SNR based on the first repetition; SNR2 = SNR based on two repetitions; Ratio = the ratio between SNR1 and SNR2; SFNR = signal to fluctuation noise ratio; RDC = radius of decorrelation; Fluc. = percent fluctuation

Figure 6 compares the QA performance between product-based and Pulseq-based protocols. For both ICE and Gadgetron reconstructions, the Pulseq-based protocol yields slightly worse absolute values for PSG, Drift, SNR_1_, and SNR_2_ compared to the product-based protocol (Fig. 6a). This discrepancy may result from differences in sequence-specific parameters, such as the fat-saturation module, spoilers, maximum gradient strength, maximum slew rate, and slice profiles of the RF pulses. Figure 6b shows the relative standard deviation differences between the two protocols, calculated as $100 \times { }\left( {\frac{{{\mathrm{STD}}_{{{\mathrm{Pulseq}}}} }}{{{\mathrm{mean}}_{{{\mathrm{Pulseq}}}} }} - { }\frac{{{\mathrm{STD}}_{{{\mathrm{Product}}}} }}{{{\mathrm{mean}}_{{{\mathrm{Product}}}} }}} \right)$. Overall, the Pulseq-based protocol demonstrates comparable relative standard deviations in QA metrics, suggesting similar day-to-day repeatability to the product-based protocol.

**Fig. 7:**
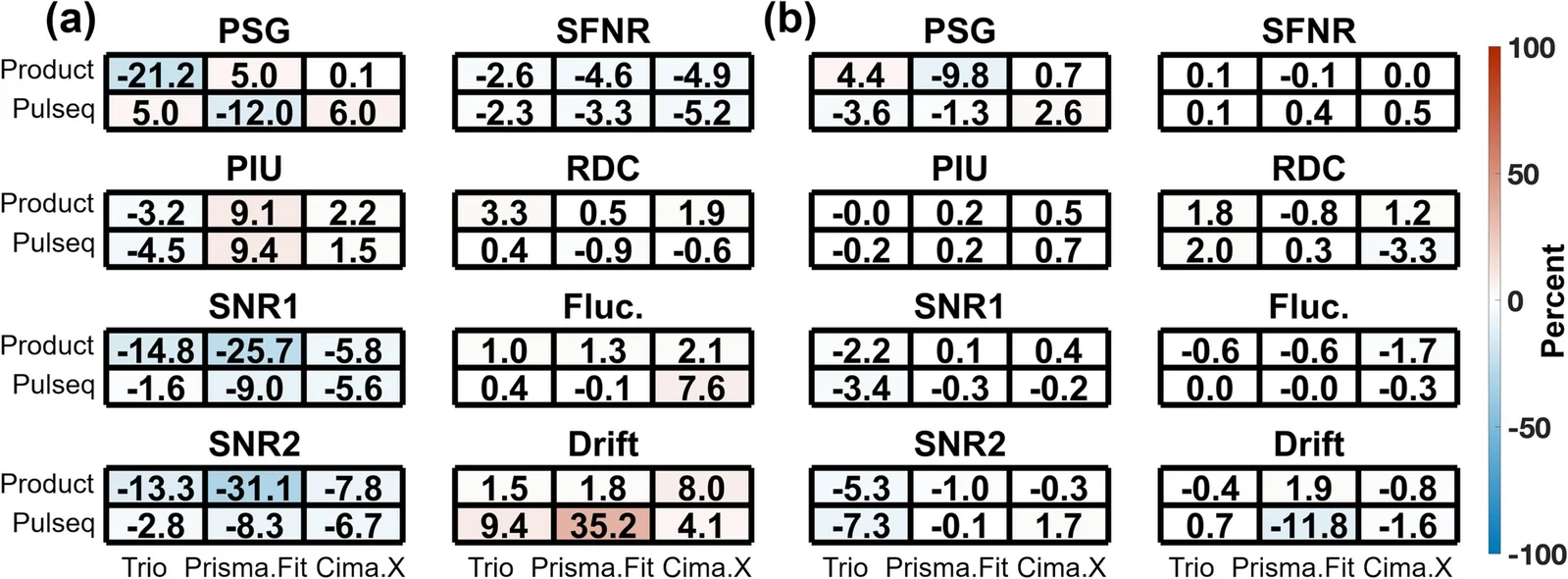
Comparison of QA performance between ICE-based and Gadgetron-based reconstructions. **a** The difference in mean QA metrics between the two reconstructions, calculated as $100 \times { }\frac{{{\mathrm{mean}}_{{{\mathrm{GT}}}} { } - {\text{ mean}}_{{{\mathrm{ICE}}}} }}{{{\text{ mean}}_{{{\mathrm{ICE}}}} }}$, for both product-based and Pulseq-based data acquisitions on the three Siemens scanners. **b** The difference in relative standard deviation, calculated as $100 \times { }\left( {\frac{{{\mathrm{STD}}_{{{\mathrm{GT}}}} }}{{{\mathrm{mean}}_{{{\mathrm{GT}}}} }} - { }\frac{{{\mathrm{STD}}_{{{\mathrm{ICE}}}} }}{{{\mathrm{mean}}_{{{\mathrm{ICE}}}} }}} \right)$, for both product-based and Pulseq-based data acquisitions on the Siemens scanners. PSG = percent signal ghosting; PIU = percent image uniformity; SNR1 = SNR based on the first repetition; SNR2 = SNR based on two repetitions; Ratio = the ratio between SNR1 and SNR2; SFNR = signal to fluctuation noise ratio; RDC = radius of decorrelation; Fluc. = percent fluctuation

Figure 7 compares the QA performance between ICE-based and Gadgetron-based reconstructions. For both product-based and Pulseq-based data acquisitions, Gadgetron produces slightly poorer absolute values for PSG, Drift, SNR_1_, and SNR_2_ compared to ICE (Fig. 7a). This difference may arise from variations in reconstruction-specific parameters, such as regridding, ghost correction, and smoothing. Figure 7b shows the relative standard deviation differences between the two reconstructions, calculated as $100 \times { }\left( {\frac{{{\mathrm{STD}}_{{{\mathrm{GT}}}} }}{{{\mathrm{mean}}_{{{\mathrm{GT}}}} }} - { }\frac{{{\mathrm{STD}}_{{{\mathrm{ICE}}}} }}{{{\mathrm{mean}}_{{{\mathrm{ICE}}}} }}} \right)$. Overall, Gadgetron provides comparable or slightly lower relative standard deviations in QA metrics, indicating similar or slightly improved repeatability across days compared to ICE.

**Fig. 8:**
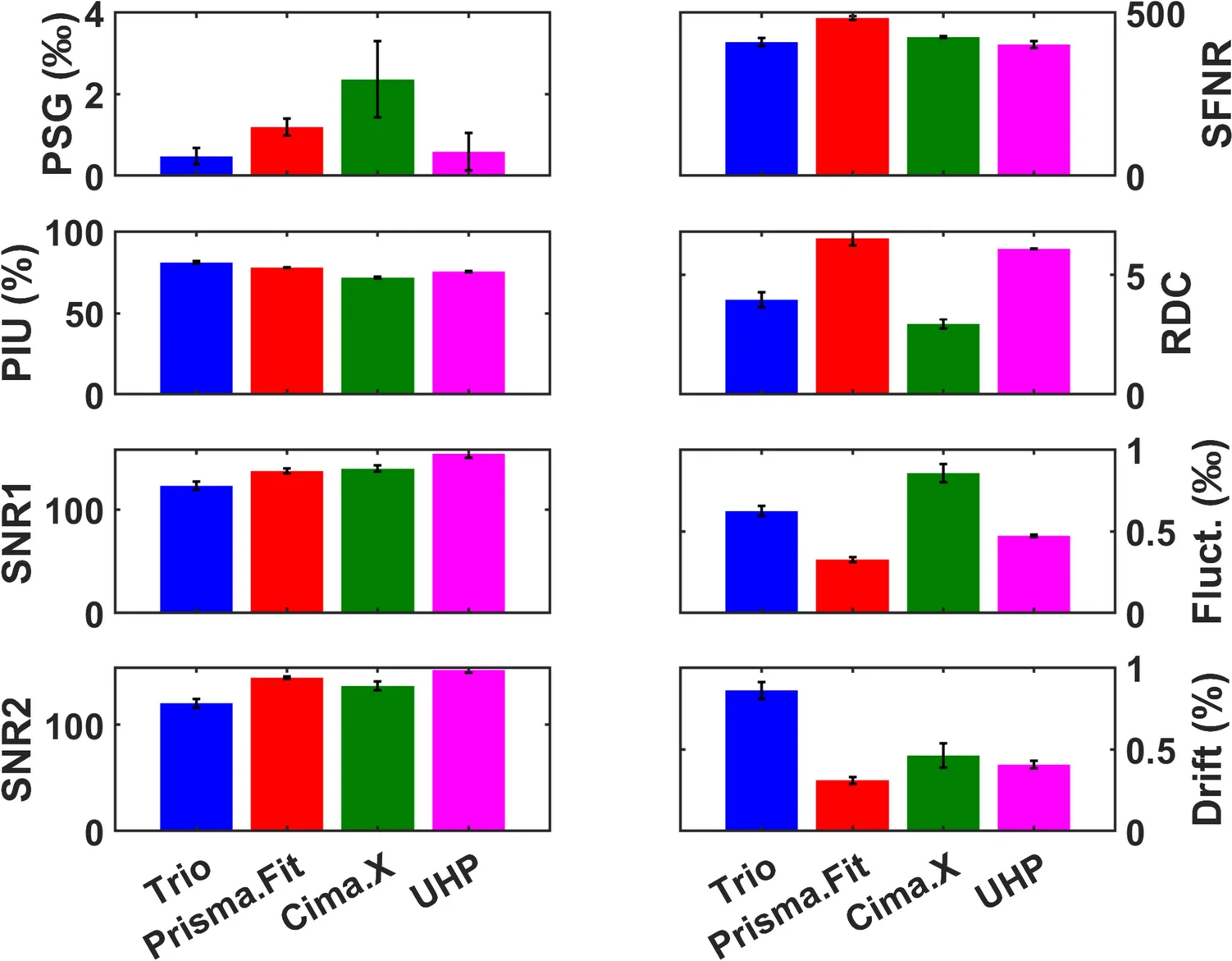
Comparison of QA performance among four scanners based on metrics calculated from Pulseq-generated, Gadgetron-reconstructed images. PSG = percent signal ghosting; PIU = percent image uniformity; SNR1 = SNR based on the first repetition; SNR2 = SNR based on two repetitions; Ratio = the ratio between SNR1 and SNR2; SFNR = signal to fluctuation noise ratio; RDC = radius of decorrelation; Fluc. = percent fluctuation

Figure 8 provides an example comparison of QA performance across the four scanners using the Pulseq-generated, Gadgetron-reconstructed metrics based on our limited number of measurements. In terms of structural quality, Trio and UHP exhibit strong PSG performance, while Cima.X ranks lowest in PSG, consistent with the prominent ghosting observed in the SE image differences (Fig. 4a). The PIU values are comparable across all scanners. In addition, UHP shows slightly higher SNR_1_ and SNR_2_ compared to the Siemens scanners, likely due to its slightly stronger B0 main field (3.0 T) compared to the Siemens systems (2.89 T). In terms of temporal quality, Prisma.Fit shows the best temporal stability, with the highest SFNR and RDC, as well as the lowest percent fluctuation and Drift, followed closely by UHP. The aging Trio scanner (17 years old) delivers the largest Drift, likely due to its age or less effective built-in compensation implemented in the hardware or low-level software. Cima.X appears to have the poorest temporal stability, as reflected by its lowest RDC and highest percent fluctuation, which correlates with the pronounced ghosting observed in the temporal fluctuation noise images (Fig. 4b).

## Discussion

Driven by the need for standardized QA in longitudinal, cross-vendor, multi-site neuroimaging studies, we developed an open-source, vendor-agnostic QA protocol. This protocol features harmonized data acquisition via Pulseq and standardized image reconstruction utilizing Gadgetron. In addition, we implemented an automated, open-source post-processing pipeline to evaluate both structural and temporal quality metrics.

Visual inspection revealed that the vendor-independent QA protocol produced images of comparable quality to those generated by the closely matched vendor-based protocol (Fig. 3). In addition, the proposed protocol yielded noise and artifact patterns similar to the vendor-based protocol (Fig. 4). Furthermore, the vendor-agnostic protocol demonstrated day-to-day repeatability comparable to that of the vendor-based protocol across the three Siemens scanners (Table 3, Figs. 6b, 7b) and exhibited high repeatability across days on the GE scanner (Table 3). These observations highlight the potential of the proposed QA package for monitoring scanner performance in longitudinal, cross-vendor neuroimaging studies.

Once long-term longitudinal QA data become available, the question inevitably arises: When does the system fall outside of acceptable control limits? Gradual changes in scanner performance may occur due to the aging of the scanner components or the Faraday cage, while sudden changes may result from factors such as software or hardware upgrades, or spikes. These underlying alterations may introduce unexplained variance into the QA data. Previous studies [22–24] have proposed statistical methods for assessing system stability in the context of manufacturing quality control. The general approach involves taking samples over time (e.g., five consecutive runs per sample) and plotting their means. The mean of a set of sample means (e.g., from five samples) and the standard error of the means (SEM) are then calculated. Confidence limits, typically set at ± 3*SEM, are applied around the overall mean. If any sample mean falls outside these limits, the system is considered beyond acceptable control limits.

The vendor-independent, repeatable QA protocol enables direct comparison of QA metrics across different scanners, vendors, and sites (Fig. 8). For example, in our evaluation with the limited number of measurements, both Trio and UHP exhibited similar PSG values that were lower than those of Prisma.Fit and Cima.X, indicating better intra-scan signal stability. The relatively high artifact levels observed in Cima.X’s SE noise images (Fig. 4a) may be attributed to eddy currents or various issues within the transmit, receive, or gradient subsystems. Cima.X also displayed the greatest temporal scanner instability during EPI scans, with the smallest RDC and the highest percent fluctuation. This instability may stem from the electrical current variations between its two gradient power amplifiers, which are responsible for driving each half of the gradient axis. In addition, the elevated ghosting levels in Cima.X’s temporal noise images (Fig. 4b) further suggest significant signal instability. Further investigation is required to determine the underlying physical causes of differences in intra-scan signal stability (PSG) and inter-scan signal stability (RDC and percent fluctuation) across different scanners and vendors.

However, the proposed QA protocol has certain limitations. For both ICE-based and Gadgetron-based reconstructions, compared to the vendor-based protocol, the Pulseq-based protocol produced poorer absolute values for PSG (notably on Trio and Cima.X), Drift (especially on Trio and Prisma.Fit), as well as SNR_1_ and SNR_2_ (Fig. 6a). The lower PSG and Drift values may be attributed to the suboptimal tuning of intricate details in the Pulseq-based SE and EPI sequences, such as the fat-saturation module, spoilers, actual used gradient strength, and actual used slew rate. The observed differences in SNR_1_ and SNR_2_ likely stem from variations in the slice excitation profiles between the vendor-based and Pulseq-based sequences. In addition, for both product-based and Pulseq-based data acquisitions, Gadgetron yielded slightly worse absolute values for PSG, Drift, SNR_1_, and SNR_2_ (Fig. 7a). This may be due to imperfect optimization of aspects of the Gadgetron-based reconstructions, such as regridding of the raw data sampled during ramps of the readout gradient and *N*/2 ghost correction. Nevertheless, the proposed protocol provided similar or slightly better QA repeatability compared to the vendor-based protocol (Figs. 6b, 7b), which secures QA data comparability across scanners, vendors, and sites, as well as over an extended period of time. Vendor-based data acquisition and image reconstruction were not performed on the GE platform; however, this does not impact direct cross-vendor QA comparisons in practice.

Another limitation lies in the calculation of the EPI ghost level, which can be affected by system instabilities over time. We found that the ghost levels were susceptible to the accuracy of the ghost region masking—misalignment could significantly overestimate ghost levels. Given the relatively small field of view and the challenges in automatically and accurately defining the ghost ROI, obtaining reliable results was difficult. To prevent potential bias from improper ghost masking, we ultimately excluded this metric from the post-processing pipeline. Future efforts should focus on refining the Pulseq-based sequences and Gadgetron-based reconstruction algorithms. In addition, expanding the QA package to include more analyses and QA metrics—such as incorporating SE and EPI sequences with parallel imaging or simultaneous multi-slice accelerations, or extending the set of protocols to include additional tests relevant to diffusion and flow MRI—could provide deeper insights into scanner performance.

Last but not least, our exploration of the QA performance characteristics across different scanners highlighted that various operational factors during data acquisition—such as shim mode, coil combination, image scaling factor, phantom temperature, and warm-up—can significantly influence the QA results. To mitigate these variations, we have established a standard operating procedure designed to enhance the comparability of QA results across different scanners, vendors, and sites. This procedure is publicly available online [15, 17].

## Conclusion

In this study, we successfully developed and implemented an open-source, vendor-independent QA protocol based on the Pulseq framework, complemented by an automated post-processing pipeline. The protocol was rigorously validated on three Siemens 3T scanners with different manufacturing dates and software versions, as well as on one GE 3T scanner. Our results demonstrate the protocol’s feasibility and confirm its repeatability across different days, system versions, vendors, and sites. These findings underscore the potential of the proposed protocol to standardize QA measures for longitudinal, cross-vendor, multi-site, large-scale neuroimaging studies.

## Data Availability

All reconstructed images and a representative raw data used in this manuscript, along with all source code and detailed instructions of the proposed QA package, are publicly available online via GitHub (https://github.com/HarmonizedMRI/qualityAssurance/tree/main) and Zenodo (https://doi.org/https://doi.org/10.5281/zenodo.14778552). Complete raw data can be acquired from the authors upon request.

## Appendix

See Table 4.

**Table 4:** Summary of all QA metrics

Type of quality	Metric	Abbreviation	Formula or definition	Image characteristics
Structural quality	Percent signal ghosting	PSG	${\mathrm{PSG}} = 100 \cdot \left| { \frac{{\left( {\overline{S}_{{{\mathrm{FE1}}}} + \overline{S}_{{{\mathrm{FE2}}}} } \right) {-} \left( {\overline{S}_{{{\mathrm{PE1}}}} + \overline{S}_{{{\mathrm{PE2}}}} } \right)}}{{2\overline{S}}}} \right|$	Signal stability across multiple TR periods during a scan
	Percent image uniformity	PIU	${\mathrm{PIU}} = 100 \cdot \left[ {1 {-} \frac{{\left( {\overline{S}_{\max } {-} \overline{S}_{\min } } \right)}}{{\left( {\overline{S}_{\max } + \overline{S}_{\min } } \right)}}} \right]$	RF or static magnetic field inhomogeneities; insufficient eddy current compensation
	Signal-to-noise ratio	SNR	${\mathrm{SNR}}_{1} = \sqrt {\left( {4 {-} \pi } \right)/2} \cdot \frac{{\overline{S}}}{{{\mathrm{SD}}_{1} }}$ ${\mathrm{SNR}}_{2} = \sqrt 2 \cdot \frac{{\overline{S}}}{{{\mathrm{SD}}_{2} }}$ ${\mathrm{SNR}}_{{{\mathrm{ratio}}}} {\text{ = SNR}}_{{1}} /{\mathrm{SNR}}_{{2}}$	Clinical utility of an MR image; hardware performance and image reconstruction efficiency; temporal stability
Temporal quality	Signal-to-fluctuation-noise ratio	SFNR	SFNR_image = signal_image/fluctuation_noise_image	Signal stability over time
	Percent fluctuation	Fluc.	100 * STD(detrended_time_series)/mean(time_series)	Signal stability over time
	Drift	–	$100 \times { }\left( {{\mathrm{trend}}_{{{\mathrm{max}}}} - {\mathrm{trend}}_{{{\mathrm{min}}}} } \right)/{\mathrm{mean(time\_series)}}$	Variations in magnetic field strength
	Radius of decorrelation	RDC	ROI side length at which the statistical independence of the pixels is lost	Signal stability over time
